# Organ dysfunction in critically ill cancer patients undergoing cytoreductive surgery with hyperthermic intraperitoneal chemotherapy

**DOI:** 10.3892/ol.2015.2921

**Published:** 2015-01-30

**Authors:** SILVIO A. ÑAMENDYS-SILVA, PAULINA CORREA-GARCÍA, FRANCISCO J. GARCÍA-GUILLÉN, HORACIO N. LÓPEZ-BASAVE, GONZALO MONTALVO-ESQUIVEL, JULIA TEXCOCANO-BECERRA, ÁNGEL HERRERA-GÓMEZ, ABELARDO MENESES-GARCÍA

**Affiliations:** 1 Department of Critical Care Medicine, National Cancer Institute, Mexico City 14080, Mexico; 2 Department of Critical Care Medicine, National Institute of Medical Sciences and Nutrition Salvador Zubirán, Mexico City 14000, Mexico; 3 Division of Education and Research, Women’s Hospital, Mexico City 11340, Mexico; 4 Division of Surgical Oncology, National Cancer Institute, Mexico City 14080, Mexico

**Keywords:** organ dysfunction, critically ill cancer patients, cytoreductive surgery, hyperthermic intraperitoneal chemotherapy, intensive care unit, critical care

## Abstract

The aim of the present study was to observe the incidence of organ dysfunction and the intensive care unit (ICU) outcomes of critically ill cancer patients during the cytoreductive surgery with hyperthermic intraperitoneal chemotherapy post-operative period. The present study included 25 critically ill cancer patients admitted to the ICU of the National Cancer Institute (Mexico City, Mexico) between January 2007 and February 2013. The incidence of organ dysfunction was 68% and patients exhibiting ≤1 organ system dysfunction during ICU admittance remained in hospital for a significantly shorter period compared with patients who exhibited ≥2 organ system dysfunctions (12.4±10.7 vs. 24.1±12.8 days; P=0.025). Therefore, the present study demonstrated that a high incidence of organ dysfunction was associated with a longer ICU hospital stay.

## Introduction

Cytoreductive surgery with hyperthermic intraperitoneal chemotherapy (HIPEC) is emerging as a favorable therapeutic strategy for the treatment of advanced, locally metastatic abdominal cavity cancer or peritoneal carcinomatosis. HIPEC is the intraoperative instillation of chemotherapeutic agents directly into the abdominal cavity and requires the use of a heating system (1).

Patients who undergo cytoreductive surgery and HIPEC typically exhibit a number of physiological changes, including cardiovascular system, oxygen consumption and coagulation effects. The most commonly observed complications include intra-abdominal sepsis, anastomotic leaks, pancreatitis, renal failure, intestinal fistulae and hematological toxicity. However, the incidence of post-operative complications is low, with ~30% of patients requiring post-operative organ support (2).

The aim of the present study was to investigate the incidence of organ dysfunction, and the clinical characteristics and intensive care unit (ICU) outcomes of critically ill cancer patients admitted to the oncological ICU of the National Cancer Institute (INCan; Mexico City, Mexico) during the cytoreductive surgery with HIPEC post-operative period.

## Patients and methods

### Patient data

The present study was an observational and descriptive investigation of 25 critically ill cancer patients admitted to the National Cancer Institute ICU during the cytoreductive and HIPEC post-operative period, between January 2007 and February 2013. Our previous studies reported data regarding patient characteristics, National Cancer Institute ICU, the cytoreductive surgery and HIPEC techniques used, and recommendations for admission requirements to the INCan ICU (3–5). HIPEC was delivered once tumor cytoreduction had been concluded. The chemotherapy solutions used were mitomycin-C (3.3 mg/m^2^/l) and cisplatin (25 mg/m^2^/l) for 90 min of perfusion (4). A roller pump forces the chemotherapy perfusion into the abdomen through a Tenckhoff catheter and pulls it out through four closed-suction drains, with a flow rate of ~1 l/min (6). A heat exchanger maintains the temperature of the fluid being infused at 43–45°C so that the intraperitoneal fluid is maintained at 41–43°C (6). Demographic, clinical and laboratory data were collected from each patient on the first day of the ICU stay during the final month prior to hospitalization, including the location of the primary tumor, comorbidity, the American Society of Anesthesiologists tumor classification grade (7), surgery duration, perioperative bleeding volume, crystalloid volume required during the surgical procedure and in the first 24 h of ICU admission, complications (medical and surgical), mechanical ventilation and vasopressor therapy requirements and duration periods, ICU and hospital length of stay, infection sites, and the ICU and hospital mortality rates. Organ dysfunction was defined by a Sequential Organ Failure Assessment (SOFA) score of ≥1 point for any organ system (5,8). Additionally, the Acute Physiology and Chronic Health Evaluation II (9) and SOFA (8) scores were calculated using the poorest acute physiological variable values determined during the first 24 h of ICU admission. Subsequently, the patients were divided into two groups according to the number of organ system dysfunctions (≤1 organ dysfunction, n=17; ≥2 organ dysfunctions, n=8). The present study was approved by the Bioethics Committee of the INCan, and the requirement for informed consent was waived.

### Statistical analysis

Categorical data are expressed as percentages and continuous variables are expressed as the mean ± standard deviation. All continuous variables were analyzed for normality using the Kolmogorov-Smirnoff test and compared by performing Student’s t-test. All statistical analyses were performed using SPSS version 21.0 software (SPSS, Inc., Chicago, IL, USA). Two-sided P<0.05 was considered to indicate a statistically significant difference.

## Results

**Figure 1:**
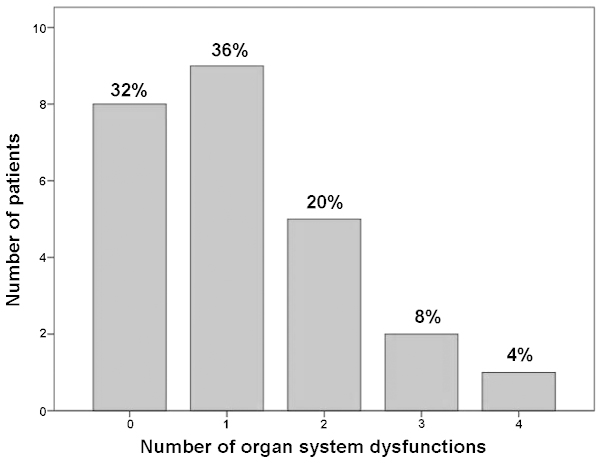
Number of organ system dysfunctions.

The mean age of the patients was 49.2±14.4 years and 76% (19 cases) were female. The ovary was the most common primary tumor site (36%) and the mean duration of surgery was 517.2±95.6 min. Furthermore, a mean blood loss volume during HIPEC of 870±732 ml was determined, with patients receiving a mean of 8.4±2.6 liters of crystalloid fluid during the surgical procedure and requiring an additional 3.7±2 liters on the first post-operative day (Table I). During the first 24 h of ICU admission, vasopressors were required by six patients (24%) for a total of 2.1±1.1 days and invasive mechanical ventilation was required by 15 patients (60%) for a median duration of one day (interquartile range, 1–2 days). Overall, seven (28%) patients required packed red blood cell transfusions. Furthermore, the incidence of organ dysfunction was 68% (17 patients; Fig. 1) and was most frequently noted in the respiratory (60%), hepatic (44%) and coagulation (28%) systems (Table II). Three (12%) patients exhibited renal dysfunction, however, no patients required or received renal replacement therapy. The mean urine output during the surgery was 64.5±31.9 ml. The length of stay in the ICU and hospital was 2.6±2 and 16.1±12.4 days, respectively; however, patients exhibiting ≤1 organ system dysfunction during their ICU stay remained in hospital for a significantly shorter period of time compared with the patients exhibiting ≥2 organ system dysfunctions (12.4±10.7 vs. 24.1±12.8 days; P=0.025) (Table III). No patients succumbed to the disease during their hospital stay.

## Discussion

HIPEC patients require large volumes of fluid, with the critical maintenance and restoration of normovolemia during the cytoreductive period necessitating the administration of solutions and blood substitutes prior to commencing the HIPEC procedure, in order to prevent excessive pathophysiological alterations occurring during the HIPEC phase (10). Previously, the administration of goal-directed fluid therapy in patients undergoing major surgery was observed to reduce renal complications, pneumonia, the time to the first bowel movement and the resumption of normal diet consumption, and the length of hospital stay, compared with non-goal-directed therapy (11). Furthermore, intraoperative fluid turnover was recorded to significantly surpass the established levels of 6–8 ml/kg/h during major abdominal interventions, with fluid loss of ≤12 ml/kg/h, depending on the degree of tumor debulking (12). In addition, Schmidt *et al* (13) reported large intra-operative fluid turnover and Arakelian *et al* (14) observed that HIPEC patients received almost 17 liters of fluid during the surgical procedure in order to maintain basic hemodynamic functions. Similarly, patients in the present study required large volumes of fluid without impacting the number of organ system dysfunctions.

In the current study, the incidence of organ dysfunction was 68% in critically ill cancer patients who were admitted to the ICU during the cytoreductive surgery and HIPEC post-operative period, with the incidence of organ dysfunction most frequently noted in the respiratory, hepatic and coagulation systems. A significant association between the number of organ dysfunctions at the time of ICU admission, and the length of stay in the hospital (P=0.025) and ICU (P=0.021) were identified. Furthermore, patients who developed ≤1 organ system dysfunction during their ICU stay remained in hospital for a significantly shorter length of time compared with the patients who exhibited ≥2 organ system dysfunctions.

Schmidt *et al* (13) reported that 66% of observed critically ill patients required ventilation upon arrival at the ICU, with a median duration of 3.7 h (range, 0.5–62.2). In the present study, 60% of observed patients required invasive mechanical ventilation; however, the majority were extubated during the first ICU day.

In the current study, 12% of patients developed renal dysfunction; however, no patients received renal replacement therapy. The volume of urine output that should be maintained during the procedure is unknown. Although Rothfield and Crowley (15) recommended a maintained level of 100 ml for every 15 min of urine output for the duration of the hyperthermic perfusion period, 50–75 ml every 15 min may be acceptable. In the current cohort, the mean urine output during the surgical procedure was 64.5 ml; this may be associated with the fact that no patients required hemodialysis during the recovery process. Similar to a study by Cooksley and Haji-Michael (2), 24% of patients in the current study required vasopressors. Thus, vasopressor support may be required for short periods during the first 24 h after ICU admission, possibly due to an acute change in body temperature and increased abdominal pressure (15).

Previously, a perioperative mortality rate ranging between 3 and 5% (16,17), and a mortality rate of 3% have been reported (16). However, no patients in the current study succumbed to the disease during their hospital stay.

The primary limitations of the present study are that it represents the experience of a single oncological ICU and that the sample size was small.

In conclusion, a high incidence of organ dysfunction was observed in the present study, with organ dysfunction associated with a longer hospital stay. HIPEC appears to be key in the treatment of cancer patients, however, upon termination of this complex procedure, critically ill cancer patients may require organ support systems to facilitate recovery in the ICU.

## Figures and Tables

**Table I:** Demographic and clinical data of the patients (n=25).

Characteristic	Value
Total patients, n (%)	25 (100)
Age, years^a^	49.2±14.4
Gender, n (%)	
Female	19 (76)
Male	6 (24)
APACHE II score^a^	6.4±4.1
SOFA score^a^	4.1±2.0
Diagnosis, n (%)	
Ovarian cancer	9 (36)
Serious	2 (8)
Mucinous	7 (28)
Pseudomyxoma	6 (24)
Colorectal adenocarcinoma	6 (24)
Others	4 (16)
Co-morbidity, n (%)	
Arterial hypertension	7 (28)
Diabetes mellitus	6 (24)
ASA classification grade, n (%)	
2	14 (56)
3	11 (44)
Duration of surgery, min^a^	517.2±95.6
Perioperative bleeding, ml^a^	870.8±732.9
Crystalloid fluid received, liters^a^	
During surgery	8.4±2.6
During the first 24 h after admission to the intensive care unit	3.7±2.0
Complications, n (%)	
Surgical	4 (16)
Medical	4 (16)

a Data presented as the mean ± standard deviation.

ASA, American society of anesthesiologists; APACHE, acute physiology and chronic health evaluation; SOFA, sequential organ failure assessment.

**Table II:** Incidence of organ system dysfunction.

Dysfunction	Cases, n (%)
Respiratory	15 (60)
Hepatic	11 (44)
Hematological	7 (28)
Cardiovascular	5 (20)
Renal	3 (12)

**Table III:** Patients according to the number of dysfunctional organ systems.

	Organ dysfunction, n^a^		
		
Variable	≤1 (n=17)	≥2 (n=8)	P-value
Length of stay, days			
ICU	2.0±1.1	4.0±3.0	0.021
Hospital	12.4±10.7	24.0±12.8	0.025
Duration of surgery, min	500.8±106.1	551.8±60.1	0.221
Crystalloid fluid recevied, liters			
During surgery	8.0±2.7	9.7±2.2	0.342
During the first 24 h after admission to the ICU	3.3±1.5	4.6±2.6	0.128
Hemoglobin, <10 g/l	12.9±1.6	10.8±2.0	0.011
Perioperative bleeding, ml	610.0±387.7	1425.0±991.1	0.007
Platelets, <100×10^9^/l	227.1±75.7	127.0±37.0	0.002
Creatinine, mg/dl	0.8±0.2	0.7±0.2	0.734
Albumin, g/dl	2.1±0.5	1.6±0.5	0.043
Lactate, mmol/l	2.3±1.0	2.6±1.5	0.550

a Data presented as the mean ± standard deviation.

ICU, intensive care unit.
